# Examining Experiences With Menstruation and Glycemic Variability: Content Analysis on Reddit

**DOI:** 10.2196/81641

**Published:** 2026-03-27

**Authors:** Kylie Lovett, Beatrice Nkunga, Haley Person, Shristi Bhochhibhoya

**Affiliations:** 1 Department of Health, Human Performance and Recreation, University of Arkansas, 155 Stadium Dr, Fayetteville, AR, 72702, United States, 1 (479) 575-2858

**Keywords:** social media, diabetes management, Reddit, menstruation, health education

## Abstract

Reddit users discussing diabetes report glycemic variability during menstruation and often turn to technology and health care for support; yet persistent gaps in provider guidance and awareness highlight the critical need for integrated care and future research at the intersection of menstrual health and diabetes management.

## Introduction

Research has demonstrated that women with diabetes experience fluctuations in glycemic control during various phases of their menstrual cycle; however, existing studies produce conflicting evidence regarding which specific phase contributes most significantly to heightened insulin resistance, ultimately leading to a variability in glycemic control and worsened quality of life due to diabetes than in men [1-3]. Social media research provides valuable insights into health-related experiences and attitudes, particularly for sensitive topics, such as menstruation [4,5]. Platforms like Reddit support anonymity and reduce social desirability bias, often yielding richer in-depth data than traditional surveys [6,7]. Since the early 2000s, engagement in Diabetes Online Communities (DOC) has continued to enable individuals with diabetes to share experiences and engage in peer advocacy [8,9]. This study aims to delve into Reddit DOC users’ experiences with managing diabetes in conjunction with their menstrual cycles, seeking to identify unique perspectives and challenges that may be overlooked in traditional survey methodologies by using five popular diabetes subreddits.

## Methods

### Overview

This study was conducted by scraping posts from the five most populous diabetes-related subreddits with approximately 13,000 to 143,000 members. We used the ExtractoR tool (ExtractoR version 3.0.8.tar.gz , R version 4.4.1) to gather data and subsequently used Microsoft Excel for data analysis. Table 1 shows the inclusion criteria, keywords, and screening process during exploration of posts associated with diabetes-related subreddits.

**Table 1.** Screening of Reddit posts based on the inclusion criteria and keywords related to menstruation.

Inclusion criteria	Keywords	Screening and final sample
Posts from five most popular subreddits of Diabetes (r/diabetes (143,000 members), r/type2diabetes (13,000 members), r/diabetes_t2 (46,000 members), r/diabetes_t1 (68,000 members), and r/Type1Diabetes (43,000 members) Posts included if they contain menstruation-related “keywords” Exclusion of any comments or links attached in the comment section All posts before Jan 1, 2025. Data extraction timeframe: Feb 1 through Feb 20, 2025	“menstruation” “menses” “Period” “Ovulation” “bleeding” “hormone” “hormones” “PCOS” “week before” “mother nature” “aunt flow” “cycle”	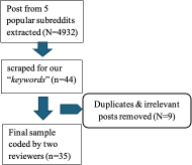

An initial codebook was developed by reviewing 20% of posts to provide a structured framework relevant to the study’s aims. Codes were refined iteratively through team discussion with a distinct definition and organized into themes and subthemes. Two coders then independently applied the finalized codebook to the full dataset. Interrater reliability was assessed using Cohen’s κ. Cohen’s κ is a statistical measure of interrater reliability commonly used in content analysis and qualitative studies to assess the agreement between two raters on categorical data [10]. Cohen’s κ value greater than 0.70 indicates acceptable reliability [10]. κ values are reported for each theme and subtheme in Table 2. Any κ values falling below the threshold of 0.70 were discussed and resolved with the assistance of a third coder, ensuring the accuracy of the coding process and strong interrater reliability.

**Table 2.** Qualitative themes and example posts associated with diabetes management and menstruation from Reddit.

Code name	Definition	Number of posts	Example posts	Cohen’s κ
Menstruation	Narratives regarding menstrual cycle, including symptoms, social and emotional experiences	27	1.1.“ *My periods are usually regular, but after my second [Trulicity] shot, my period came about a week early.”* 1.2. *“I’m on Mounjaro and Metformin, for both it’s the second week and my period is late*.	0.72
1a. Emotional effects	Self-report or other’s acknowledgment of emotional, psychological, or mental changes that happen during different phases of the menstrual cycle, including anger, sadness, irritability, relief of getting period, told by partner they are acting different, etc	9	1a.1. *“I feel so depressed everyday. I can’t sleep, I have anxiety, and my self esteem is extremely low. At times I feel like I hate myself and I feel like I will never be the same after my diabetes diagnosis. ”* 1a.2. *“does anyone else experience PMS^a^ even without a period? . I definitely experience PMS (slight bloating, much more sensitive to noises, and I’m much more irritable).”*	1.00
1b. Physical effects	Self-report of bodily sensations that happen during or around the time of menstruation. May include headache, cramps, change in appetite, nausea, breast tenderness etc Includes being tired, migraines, not being able to attend work/school, nausea, vomiting, unable to eat, eating disorders	14	1b.1.*“ Since starting insulin treatment, I’ve gained a considerable amount of weight., My period is now irregular every month and I have hot flashes and my acne has flared up.”* 1b.2.*“And my period is incredibly painful with really, really heavy flow*. *I feel weak overall, it was moderate/normal before.”*	0.87
1c. Irregular periods	Self-report of abnormal symptoms of period, such as longer period days, multiple periods in one month, longer menstrual cycle, abnormal spotting, heavy flow, no flow, too frequent or infrequent periods	12	1c.1.*“My periods starting becoming less frequent last year, and finally stopped altogether.”* 1c.2.*“ since starting insulin treatment,my period is now irregular every month and sometimes I don’t get it at all.”* 1c.3.*“I originally found out I have diabetes because I hadn’t gotten my period for almost three months,...”*	0.80
1d. Other hormonal comorbidities	Mentions being told by a health professional they have other medical conditions that coexist with menstruation and diabetes and impact hormonal health. Includes PCOS^b^, Hashimoto’s, thyroid disorders, but not cold, flu, stress, obesity	3	1d.1.*“I was diagnosed with Type 1 almost 30 years ago and with PCOS recently ,*	0.79
Management	Any mentions of strategies, routines, tools or behavior used to monitor, control, or adapt to the effects of menstruation on diabetes. Includes posts that mentions Hba_1c_ values, diet exercise, medications controlling glucose, trial and error, lived experiences. Does not include just symptoms and challenges without references to how they respond or manage them.	21	2.1.“*Being 270+ for days now, been drinking honey to get better so it messes up with my glucose, too tired to go for walks.”* 2.2.“*I only had a small smoothie this morning and no lunch. Dinner comes around (literally only a few things), and I take an extra 2 units [of insulin] than I would normally, and I’m STILL spiking”*	0.80
2a. Technology	Digital tools, apps, or platforms used to support the management of diabetes. Includes mentions of Insulin pumps (Tandum, Omnipod, Minimed, Medtronic 670G, 660G, 770G, 780G) Continuous Glucose Monitors (CGM, Dexcom, Libre, Freestyle Libre, Libre 6, Guardian, sensor), closed loop systems (usually with a Tandum and Omnipod (O5)), Automated Insulin Delivery Systems (AIDs) other key words include cannula, port, site, infusion set, wires, pump, charts showing glucose numbers, AI pancreas, telehealth, phone apps, frustrations with technology, social media support groups, smart insulin pens.	5	2a.1.“*I have a CGM and recently got a pump which has made a huge difference, but I still struggle to manage my glucose levels.”* 2a.2.*“[The user’s daughter] is on the O5 (Omnipod 5) and we’ve already lowered her carb ratios, but, still, she goes low. ”*	1.00
2b. Medical team	References to health care professionals or clinical teams, such as doctors, nurses, PC^c^, DNP^d^, who are involved in the management of diabetes and/or menstruation. This includes both negative and positive experiences or perceptions of while communicating with providers.	11	2b.1. *“My doctor just sent in the prescription. This was after monitoring sugars and trying to lose weight in order to conceive. ”* 2b.2. *“Doc told me how proud he was of me for turning it around so fast in such a short time. ”* 2b.3. *“Diagnosed type 2 diabetic and my doctor increased me from 1 pill of Metformin ER 750mg to 2 pills due to my A1C going up ”* 2b.4.*“I did visit an endocrinologist once for my irregular periods, but they wanted to put me on birth control, which I wasnt comfortable with.”* 2b.5.*“ My previous OBGYN and pharmacist scared me out of the contraceptiove shot last year and were trying to push the Mirena IUD on me which I am not comfortable with. ”*	1.00

a PMS: Pre-menstrual Syndrome.

b PCOS: Polycystic Ovary Syndrome.

c PC: Primary Care.

d DNP: Doctor of Nursing Practice.

### Ethical Considerations

The University of Arkansas Institutional Review Board classified this study as nonhuman subjects research and exempt from review for involving secondary analysis of existing public data. No identifiable information, such as usernames, timestamps, or links, of participants in public subreddit groups was analyzed. Users’ identities were further protected by shortening, masking highly identifiable details, and paraphrasing by substantial alteration of wording and structure of posts to minimize risks of reidentification. Data were stored on secure, access-restricted university servers. This study was supported by the University of Arkansas Honors College.  

## Results

Table 1 presents predefined keyword-based inclusion criteria that identified 35 unique posts regarding glycemic control and menstruation.

### Theme 1: Menstruation

Among these 35 posts, 27 posts shared characteristics of the users’ menstrual cycles and any alterations they noticed in their menstruation due to diabetes. Many users reported changes in their menstrual cycles following their diabetes diagnosis. Some experienced regular cycles for the first time, while others noted alterations in the frequency or characteristics of their periods, such as changes in flow and cycle length, as shown in example posts in Table 2. Four distinct subthemes emerged: emotional effects, physical effects, irregular periods, and other hormonal comorbidities, all showing strong interrater reliability.

### Theme 2: Management Strategies

A total of 21 posts discussed management strategies, various routines, behaviors, and tools designed to mitigate the impact of menstruation on diabetes control. Many users reported engaging in physical activity or administering insulin when experiencing elevated blood sugar levels and adjusting their dietary habits according to their menstrual cycle phases. Table 2 shows two key subthemes: Technology and Medical Team, with strong interrater reliability.

## Discussion

Most Reddit users discussed menstruation and diabetes management strategies, particularly blood glucose monitoring and control across menstrual cycle phases. Many described experiencing a range of physical and emotional symptoms while undergoing irregular, prolonged, or frequent periods. Although they did not specify the most challenging phase of the menstrual cycle for glucose control, they noted persistent elevation in blood glucose during a certain phase that affected their physical health (eg, bloating) and mental well-being (eg, irritability). These reports of emotional distress are consistent with prior research demonstrating that cyclical hormonal fluctuations can increase insulin resistance and glycemic variability, creating periods of unpredictability that place significant cognitive and emotional burdens on individuals managing diabetes [1-3].

Most users reported challenges of diabetes management during menstrual hormonal changes, along with the co-occurrence of other hormonal disorders, such as polycystic ovarian syndrome and thyroid disease. Furthermore, users shared diverse management strategies to address their diabetes, ranging from leveraging technology to seeking support from medical professionals. Some users’ posts referenced consultations with medical professionals regarding diabetes and menstruation-related issues, but did not provide further details on the perceived benefits of these interactions. However, some users report insufficient guidance from health care providers on managing the intersection of diabetes and menstrual abnormalities, contributing to mental stress and reinforcing prior evidence that gaps in clinical guidance led to trial-and-error in diabetes management.

This study is one of a limited body of work that examines Reddit posts at the intersection of people’s menstrual and diabetes management experiences, and some limitations are to be noted. Anonymous posts from Reddit users discussing menstruation and diabetes limit our ability to explore the gender identity and reproductive stages of the users, thereby restricting the generalizability of the results. Additionally, this study offers a snapshot of posts, eliminating the comments associated with them that were publicly available at the time of the analysis.

Our findings reveal a complex relationship between menstruation and diabetes management discussed by Reddit users, highlighting challenges in managing the intersecting hormonal conditions, which may imply a greater need for menstruation-informed care. Future studies should delve deeper into people’s personal experiences relating to diabetes and menstruation, and how they can be supported with comprehensive health education to manage their health challenges associated with menstruation and diabetes management.

## Abbreviations

DOC: Diabetes Online Communities
PCOS: polycystic ovarian syndrome
